# Ligand co-crystallization of aminoacyl-tRNA synthetases from infectious disease organisms

**DOI:** 10.1038/s41598-017-00367-6

**Published:** 2017-03-16

**Authors:** Spencer O. Moen, Thomas E. Edwards, David M. Dranow, Matthew C. Clifton, Banumathi Sankaran, Wesley C. Van Voorhis, Amit Sharma, Colin Manoil, Bart L. Staker, Peter J. Myler, Donald D. Lorimer

**Affiliations:** 1 Seattle Structural Genomics Center for Infectious Disease (SSGCID), Bethesda, MD USA; 2 Beryllium Discovery Corp, Bainbridge Island, WA, 98110 USA; 3 Berkeley Center for Structural Biology, Advanced Light Source, Berkeley, CA 94720 USA; 4 University of Washington, Seattle, WA 98195-6423 USA; 5 International Center for Genetic Engineering and Biotechnology, New Delhi, 110 067 India; 6 University of Washington, Department of Genome Sciences, Seattle, WA 98195-5065 USA; 7 Center for Infectious Disease Research (formerly Seattle Biomedical Research Institute), Seattle, WA 98109 USA; 8 University of Washington, Department of Medical Education and Biomedical Informatics & Department of Global Health, Seattle, WA 98195 USA

## Abstract

Aminoacyl-tRNA synthetases (aaRSs) charge tRNAs with their cognate amino acid, an essential precursor step to loading of charged tRNAs onto the ribosome and addition of the amino acid to the growing polypeptide chain during protein synthesis. Because of this important biological function, aminoacyl-tRNA synthetases have been the focus of anti-infective drug development efforts and two aaRS inhibitors have been approved as drugs. Several researchers in the scientific community requested aminoacyl-tRNA synthetases to be targeted in the Seattle Structural Genomics Center for Infectious Disease (SSGCID) structure determination pipeline. Here we investigate thirty-one aminoacyl-tRNA synthetases from infectious disease organisms by co-crystallization in the presence of their cognate amino acid, ATP, and/or inhibitors. Crystal structures were determined for a CysRS from *Borrelia burgdorferi* bound to AMP, GluRS from *Borrelia burgdorferi* and *Burkholderia thailandensis* bound to glutamic acid, a TrpRS from the eukaryotic pathogen *Encephalitozoon cuniculi* bound to tryptophan, a HisRS from *Burkholderia thailandensis* bound to histidine, and a LysRS from *Burkholderia thailandensis* bound to lysine. Thus, the presence of ligands may promote aaRS crystallization and structure determination. Comparison with homologous structures shows conformational flexibility that appears to be a recurring theme with this enzyme class.

## Introduction

During protein synthesis aminoacylated tRNAs bind to the ribosome with the anticodon loop pairing with the codon of the mRNA template while delivering the incoming amino acid to the elongating polypeptide. Aminoacyl-tRNA synthetases (aaRSs or aminoacyl tRNA ligases) charge tRNAs with their cognate amino acids in a two-step mechanism^1^. First, the aaRS combines the specific amino acid with adenosine-5′-triphosphate (ATP) to produce an activated aminoacyl-adenylate intermediate which reacts with the appropriate tRNA to produce the aminoacylated tRNA. Inhibition of either step results in the buildup of uncharged tRNAs in the cell and consequently on the ribosome thereby inhibiting protein synthesis^2^. In general, aaRSs are divided into two classes based on the global fold and sequence conservation^3^. The active sites of class I aaRSs contain a Rossmann fold with two highly conserved sequence motifs, HIGH and KMSKS. The active sites of class II aaRSs contain an anti-parallel β-sheet. Class I aaRSs recognize the CCA acceptor stem by approaching via the minor grove, whereas class II aaRss recognize the CCA acceptor stem via the major groove, a recognition strategy similar to an *in vitro* selected aminoacyl tRNA synthetase ribozyme^4^. Each class is further divided into three subclasses based on subunit structure and sequence conservation. Significant differences have been noted between the prokaryotic and eukaryotic homologs in several aaRSs, implying that these enzymes may be viable candidates for antimicrobial drugs^2, 5^. Indeed, the methicillin-resistant *Staphylococcus aureus* (MRSA) IleRS inhibitor mupirocin has been approved for clinical use, and its binding site has been shown by X-ray crystallography to overlap with the Ile-AMP reactive intermediate^6, 7^. Due to the presence of an ester bond which is rapidly hydrolyzed in blood plasma, mupirocin is limited to topical use.

A number of aaRS inhibitors are in preclinical development^2^. These include natural products such as borrelidin that targets a number of ThrRSs through an allosteric mechanism and ochratoxin A that targets PheRS as an active site inhibitor. Structures have not yet been determined for either of these inhibitors bound to their target aaRS, although modeling studies based on resistance mutations have provided insight into the putative borrelidin binding site^8^. In contrast, a number of structures have been published for aminoacyl-adenylate reactive intermediate analogs, such as sulfonamides^9–12^. However, these compounds typically also inhibit the human homolog and therefore have been abandoned as drug candidates. More recently, a series of diamino quinoline compounds has been developed against Gram-positive bacterial MetRSs, first by GlaxoSmithKline^13^, then by Replidyne^14–16^, and an academic group^5, 17^. These compounds exhibit strong selectivity for the bacterial MetRS over human MetRS, but may suffer from poor bioavailability^5^. Therefore, further research is necessary both in lead development and aaRS structural biology. The crystal structures of *P. falciparum* LysRS and ProRS with cladosporin or halofuginone represent valuable studies of aaRS complexes with nature product-like anti-malarial inhibitors^18, 19^.

Due to their biological importance and potential as therapeutic targets, aminoacyl-tRNA synthetases have been targeted by a number of structural genomics centers. Perhaps the most successful structural genomics centers at studying aaRSs has been the Medical Structural Genomics of Pathogenic Protozoa (MSGPP), which along with subsequent efforts has resulted in nearly twenty aaRS crystal structures^17, 20–25^. Nearly one hundred aaRSs have entered the Seattle Structural Genomics Center for Infectious Disease (SSGCID)^26–28^ structure determination pipeline, both as internally selected targets and also as targets nominated by the scientific community. These targets are largely comprised of aaRSs from Gram-negative bacteria such as *Borrelia burgdorferi*, which causes Lyme disease^29^; *Brucella melitensis*, which causes brucellosis or Malta fever; orthologs of *Mycobacterium tuberculosis*, which causes tuberculosis; and *Rickettsia prowazekii*, the etiologic agent of epidemic typhus. Other targets include a smaller number of aaRSs from eukaryotic pathogens such as *Ehrlichia chaffeensis* and *Encephalitozoon cuniculi*. Several aaRSs from *Burkholderia thailandensis* were identified as candidate essential genes in a transposon screen^30^. A number of these selected aaRS enzymes have been successfully purified, although none of them reached structure determination through first pass pipeline techniques. Here we describe our efforts to obtain aminoacyl-tRNA synthetase structures from infectious disease organisms, which have resulted in six new aaRS co-crystal structures; the initial structure of a seventh target identified via this strategy was recently reported along with inhibitor complexes^31^. All of these structures contain a ligand which may be important for stabilizing the enzyme and promoting crystallizability.

## Results and Discussion

### Co-crystallization of aaRSs from SSGCID organisms

For the initial round of crystallization of SSGCID targets, no ligands were added to the protein solution, and in general two crystallization trials were set up in 96-well format most commonly in the JCSG+ and PACT sparse matrix screens^32^, although depending on the day-to-day availability one or both of these screens were substituted with Wizard III/IV, Wizard I/II, CSHT or Morpheus. If diffraction quality crystals were not obtained from the initial round of crystallization trials, 6–8 additional sparse matrix trials were set up in 96-well format for high value targets such as those requested by the scientific community. During this second round, the protein concentration was adjusted depending on the percentage of drops containing precipitation in the first round (aiming for approximately 30–50% precipitation as optimal). During the third round of crystallization, a subset of the available aaRS protein samples were incubated with 5 mM ATP (Sigma-Aldrich) and 5 mM of the cognate amino acid (Sigma-Aldrich), and four additional sparse matrix screens were initiated, typically JCSG+, PACT, Wizard III/IV and CSHT. The combined results for the first three rounds of crystallization trials are shown in Table 1. Of the 31 proteins selected for co-crystallization trials, 18 produced crystals (58%), 11 produced crystals which diffracted to better than 6 Å resolution (35%), and 7 crystal structures were determined (23%). Overall, these rates are comparable with other protein classes in the SSGCID pipeline. X-ray diffraction data and structure determination statistics for the six structures are shown in Table 2 and the individual structures are detailed below. Although we solved one or more structures of most aaRS subclasses, we were unable to obtain a co-crystal structure of subclass 2c, perhaps in part due to the low solubility of L-phenylalanine or ochratoxin A in aqueous solution at crystallography concentrations.

**Table 1:** Co-crystallization of aaRSs from SSGCID organisms.

Class	aaRS	Organism	TargetDB ID	Ligand	Crystals	Diffraction	Structure
Ia	ArgRS	*Brucella melitensis*	BrabA.00164.a	Arg/ATP	✓	✓	
Ia	CysRS	*Anaplasma phagocytophilum*	AnphA.00302.a	Cys/ATP	✓		
	CysRS	*Bartonella henselae*	BaheA.00133.a	Cys/ATP			
	CysRS	*Borrelia Burgdorferi*	BobuA.00133.a	Cys/ATP	✓	✓	✓
	CysRS	*Brucella melitensis*	BrabA.00133.a	Cys/ATP			
	CysRS	*Burkholderia pseudomallei*	BupsA.00133.a	Cys/ATP			
	CysRS	*Rickettsia prowazekii*	RiprA.00133.a	Cys/ATP	✓	✓	
Ia	MetRS	*Anaplasma phagocytophilum*	AnphA.10201.a	SeMet/ATP			
	MetRS	*Bartonella henselae*	BaheA.10201.a	SeMet/ATP			
	MetRS	*Brucella melitensis*	BrabA.10201.a	SeMet/ATP	✓	✓	✓
	MetRS	*Burkholderia pseudomallei*	BupsA.10201.a	SeMet/ATP			
Ib	GluRS	*Borrelia Burgdorferi*	BobuA.01348.a	Glu	✓	✓	✓
	GluRS	*Burkholderia thailandensis*	ButhA.01187.a	Glu	✓	✓	✓
	GluRS	*Ehrlicia chaffensis*	EhchA.01521.a	Glu			
Ic	TrpRS	*Anaplasma phagocytophilum*	AnphA.00430.a	Trp/ATP	✓		
	TrpRS	*Bartonella henselae*	BaheA.00241.a	Trp/ATP	✓		
	TrpRS	*Encephalitozoon cuniculi*	EncuA.00600.a	Trp/ATP	✓	✓	✓
Ic	TyrRS	*Anaplasma phagocytophilum*	AnphA.01028.a	Tyr/ATP	✓	✓	
	TyrRS	*Borrelia Burgdorferi*	BobuA.01032.a	Tyr/ATP	✓		
	TyrRS	*Encephalitozoon cuniculi*	EncuA.00932.a	Tyr/ATP	✓		
IIa	HisRS	*Burkholderia pseudomallei*	BupsA.00063.a	His/ATP	✓		
	HisRS	*Burkholderia thailandensis*	ButhA.00063.a	His/ATP	✓	✓	✓
	HisRS	*Ehrlicia chaffensis*	EhchA.00686.a	His/ATP			
IIa	ThrRS	*Bartonella henselae*	BaheA.10252.a	Borrelidin			
	ThrRS	*Brucella melitensis*	BrabA.000156.a	Borrelidin			
	ThrRS	*Burkholderia pseudomallei*	BupsA.00156.a	Borrelidin	✓	✓	
IIb	LysRS	*Burkholderia thailandensis*	ButhA.00612.a	Lys	✓	✓	✓
IIc	PheRS	*Brucella melitensis*	BrabA.00163.a	Phe/ATP	✓		
	PheRS	*Mycobacterium abscesus*	MyabA.00163.a	Phe/ATP			
	PheRS	*Mycobacterium marinum*	MymaA.00163.a	Phe/ATP			
	PheRS	*Mycobacterium smegmatis*	MysmA.00163.a	Phe/ATP

**Table 2:** X-ray diffraction data and structure determination statistics.

aaRS	CysRS	GluRS	GluRS	TrpRS	HisRS	LysRS
TargetDB	BobuA.00133.a	BobuA.01348.a	ButhA.01187.a	EncuA.00600.a	ButhA.00063.a	ButhA.00612.a
Organism	*Borrelia burgdorferi*	*Borrelia burgdorferi*	*Burkholderia thailandensis*	*Encephalitozoon cuniculi*	*Burkholderia thailandensis*	*Burkholderia thailandensis*
Class^a^	Ia	Ib	Ib	Ic	IIa	IIb
Ligand	AMP, Zn^2+^	L-glutamic acid, Zn^2+^	L-glutamic acid	L-tryptophan	L-histidine	L-lysine
*Data collection*						
Beamline	ALS 5.0.1	Rigaku SuperBright FR-E+	ALS 5.0.1	CLS 08ID-1	ALS 5.0.3	Rigaku SuperBright FR-E+
Wavelength (Å)	0.97740	1.5418	0.9774	0.97949	0.97684	1.5418
*Data reduction*						
Space Group	*P*2_1_	*P*2_1_2_1_2_1_	*P*4_1_2_1_2	*P*2_1_2_1_2_1_	*P*2_1_2_1_2_1_	*P*2_1_
Unit Cell	*a* = 62.62 Å, *b* = 49.89 Å, *c* = 179.63 Å, α = γ = 90°, β = 93.18°	*a* = 61.46 Å, *b* = 110.31 Å, *c* = 197.88 Å, α = β = γ = 90°	*a* = *b* = 88.95 Å, *c* = 132.27 Å, α = β = γ = 90°	*a* = 54.11 Å, *b* = 79.16 Å, *c* = 177.01 Å, α = β = γ = 90°	*a* = 70.16 Å, *b* = 116.36 Å, *c* = 142.99 Å, α = β = γ = 90°	*a* = 86.22 Å, *b* = 118.54 Å, *c* = 94.54 Å, α = γ = 90°, β = 113.23°
Solvent content (%)	48.8	56.6	48.8	39.7	56.2	67.0
V_m_ (Å^3^/Da)	2.40	2.83	2.40	2.04	2.81	3.73
Resolution (Å)	50–2.55 (2.62–2.55)^b^	50–2.60 (2.66–2.60)	50–2.05 (2.10–2.05)	50–2.6 Å (2.67–2.60)	50–2.65 Å (2.72–2.65)	50–2.4 Å (2.46–2.40)
I/σ	12.5 (2.5)	10.2 (3.4)	24.9 (4.7)	14.7 (2.2)	24.3 (4.2)	10.9 (2.3)
Completeness (%)	99.5 (99.0)	97.0 (88.9)	100 (100)	96.5 (89.5)	99.5 (99.9)	98.1 (96.1)
R_merge_	0.107 (0.540)	0.107 (0.374)	0.065 (0.505)	0.078 (0.517)	0.065 (0.525)	0.080 (0.468)
Multiplicity	4.6 (4.1)	5.3 (4.2)	14.3 (9.2)	4.8 (3.4)	7.8 (5.4)	2.8 (2.4)
Reflections	36,598 (2741)	41,054 (2762)	34,013 (2455)	23,335 (1575)	34,582 (2541)	67,117 (11,805)
Mosaicity	0.3	1.1	0.4	0.9	0.8	0.7
*Refinement*						
R	0.227 (0.304)	0.226 (0.263)	0.201 (0.220)	0.204 (0.271)	0.208 (0.330)	0.191 (0.281)
R_free_	0.274 (0.365)	0.285 (0.316)	0.237 (0.265)	0.247 (0.386)	0.240 (0.407)	0.223 (0.331)
r.m.s.d. bonds (Å)	0.011	0.002	0.011	0.009	0.012	0.012
r.m.s.d. angles (°)	1.538	0.682	1.427	1.392	1.464	1.444
Mean *B*-factors (Å^2^)	27.3	37.5	34.6	43.2	51.8	33.0
Ligand *B*-factors (Å^2^)	35.1	24.2	23.8	37.4	42.1	23.3
*Validation*						
Ramachandran Favored (%)	96.7	97.4	97.9	97.2	98.6	98.7
Ramachandran Allowed (%)	99.6	99.8	100	99.7	100	99.9
Molprobity^55^ Score	2.24	1.39	1.10	2.24	1.78	1.42
PDB ID	3SP1	4GRI	4G6Z	3TZE	4E51	4EX5

^a^Class I aaRS enzymes contain a Rossman fold and class II aaRS enzymes contain an anti-parallel b-sheet. Additional differences are described^3^.

^b^Values in parenthesis indicate the highest resolution shell. 20 shells were used in XSCALE^56^.

### CysRS from Borrelia burgdorferi bound to AMP

**Figure 1:**
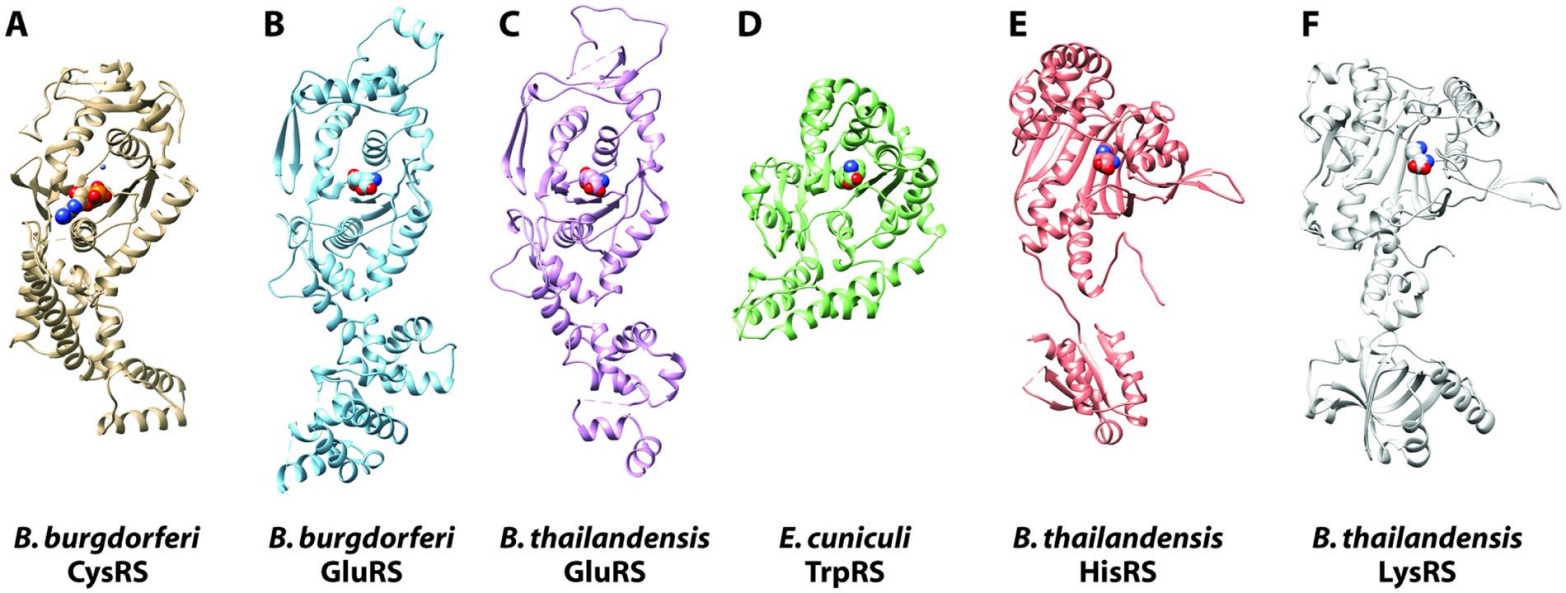
Overview of co-crystal structures of aaRS enzymes from infectious disease organisms. In the current study, we have determined 6 co-crystal structures of aminoacyl tRNA synthetase (aaRS) enzymes from infectious disease organisms: CysRS from *Borrelia burgdorferi* (**A**), GluRS from *B. burgdorferi* (**B**) and *Burkholderia thailandensis* (**C**), TrpRS from Encephalitozoon cuniculi (**D**), HisRS from *B. thailandensis* (**E**), and LysRS from *B. thailandensis* (**F**). For sake of simplicity, only a single monomer is shown although some are biological oligomers such as HisRS which is a dimer.

**Figure 2:**
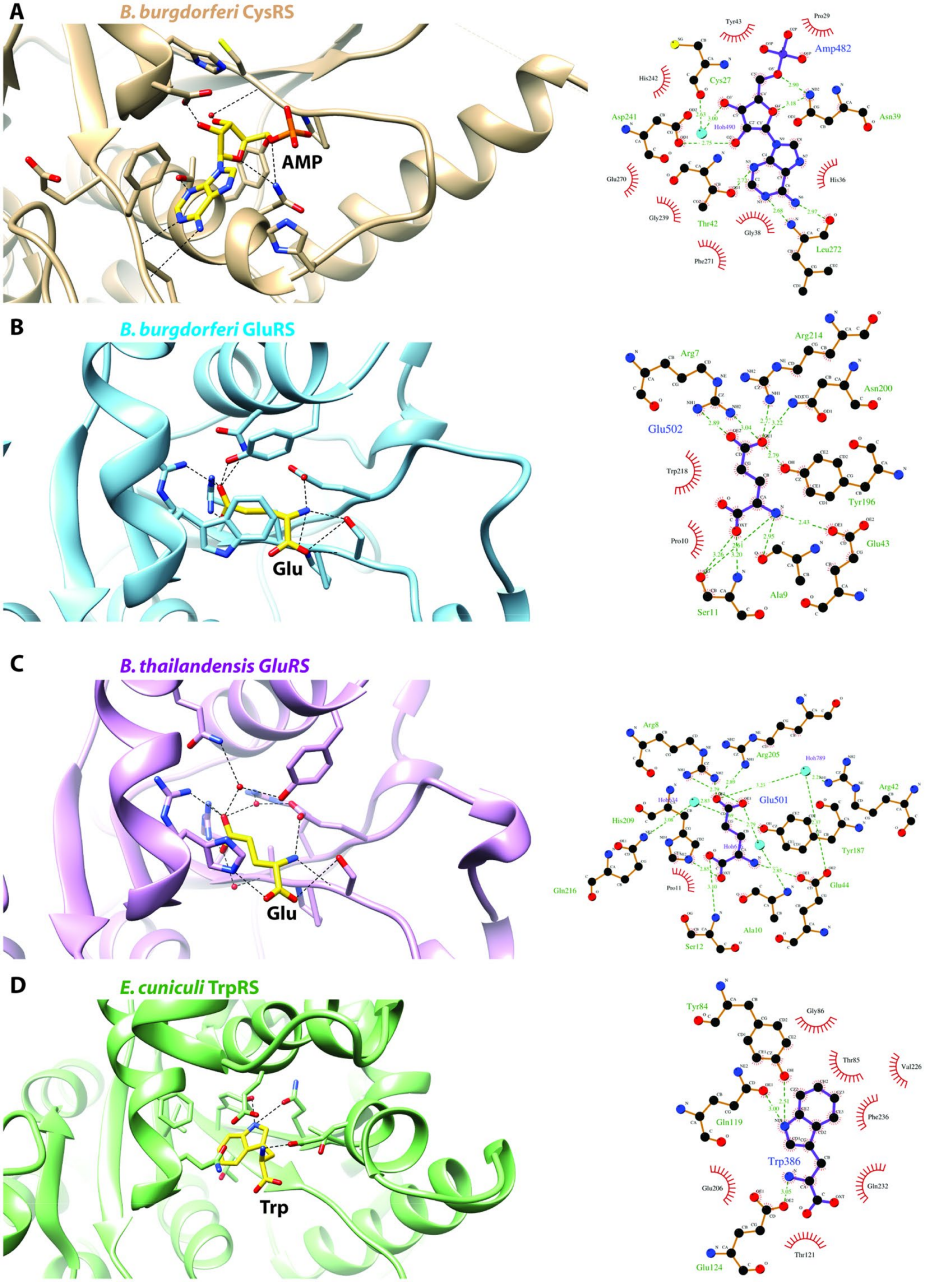
Ligand recognition by class 1 aaRS enzymes from infectious disease organisms. (**A**) class 1a CysRS from *Borrelia burgdorferi* (**B**) class 1b GluRS from *B. burgdorferi* (**C**) class 1b GluRS from *Burkholderia thailandensis* and (**D**) class 1c TrpRS from *Encephalitozoon cuniculi*.

**Figure 3:**
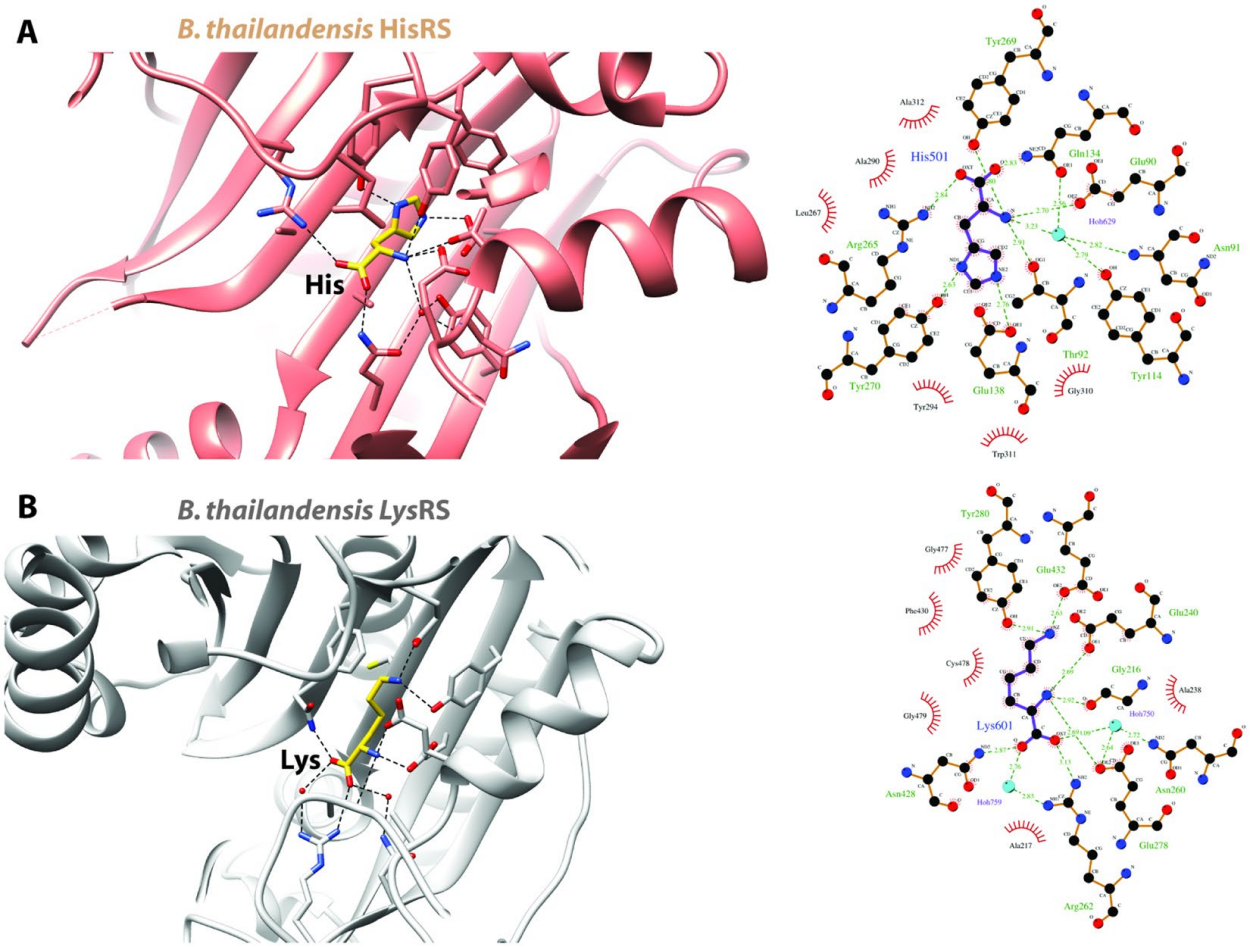
Ligand recognition by class 2 aaRS enzymes from infectious disease organisms. (**A**) Class 2a HisRS from *B. thailandensis* and (**B**) class 2b LysRS from *B. thailandensis*.

Crystal structures of cysteinyl-tRNA synthetase (CysRS, E.C. 6.1.1.16) from *E. coli* have been reported as apo, bound to substrate^33^, and in complex with tRNA^34^. Interestingly, in some organisms a CysRS enzyme has not been identified, and a prolyl-tRNA synthetase (ProRS) exhibits cross-reactivity to charge tRNAs with cysteine, although this may represent mis-acylation rather than a truly bifunctional enzyme^10^. A structure of human CysRS has not yet been solved. We determined a 2.55 Å resolution structure of CysRS, a class Ia aaRS, from *B. burgdorferi*, the causative agent of lyme disease^29^ (Figs 1A and 2A). For each of the six co-crystal structures determined here, a view of the full monomeric structure for each aaRS is shown in Fig. 1. The active sites of each aaRS are highlighted in Fig. 2 for class I aaRSs and in Fig. 3 for class II aaRS. This was the second organism for which a CysRS structure has been reported, although the structure of CysRS from *Coxiella burnetti* has now been reported (PDB ID 3TQO^35^) with RMSD 1.46 Å and sequence homology of 34%. The *B. burgdorferi* CysRS structure was solved during the second round of crystallization trials for this target, as detailed above. The central catalytic domain of CysRS is quite similar between the *E. coli*, *B. burgdorferi*, and *C. burnetti* CysRSs catalytic domains, although the C-terminal anti-codon recognition domain adopts dramatically different conformations with respect to the catalytic domain. The *E. coli* CysRS cystine and zinc bound structure (1LI7)^33^ had a backbone RMSD of 1.61 Å compared to the *B. burgdorferi* CysRS structure. After determining the structure of CysRS from *B. burgdorferi*, initial inspection of the electron density maps revealed two strong difference density features. The first difference density peak most likely corresponded to the catalytic zinc ion, as inferred by the *E. coli* homolog and which modeled and refined appropriately. The second strong difference density was supportive for an AMP or AMP-containing molecule, which resides in the same location as the A of the CCA tail of (site of acylation) in the *E. coli* CysRS/tRNA^Cys^ crystal structure. Due to additional residual density off the phosphate of the AMP, it appears likely that a mixture of AMP-containing compounds may have co-purified from the expression host or represent a mixed population of degraded or disordered ATP, which was added during co-crystallization. Attempts to co-crystallize with tRNA mini-helices containing the CCA acceptor stem were unsuccessful.

### *GluRS from* Borrelia burgdorferi *and* Burkholderia thailandensis *bound to L-glutamic acid*

A number of glutamyl-tRNA synthetase (GluRS E.C. 6.1.1.17) crystal structures have been reported in the literature from bacteria, eukaryotes, and archaea. Unfortunately, the human structure has yet to be solved by X-ray crystallography. We solved two co-crystal structures of the class Ib GluRS bound to L-glutamic acid, one from B. burgdorferi at 2.6 Å resolution and one from B. thailandensis at 2.05 Å resolution (Figs 1B,C and 2B,C). Differences between the two GluRS structures in the cognate amino acid binding pocket are apparent. For example, in the B. thailandensis GluRS structure, His209 makes a hydrogen bond with the main chain carboxylate of the cognate glutamic acid, but a hydrogen bond is not observed from the equivalent Trp residue in the B. burgdorferi structure. In the B. thailandensis GluRS structure several water-mediated interactions were observed in the amino acid binding pocket compared to the B. burgdorferi GluRS structure, presumably due to the higher resolution of the B. thailandensis GluRS structure. The two structures have an RMSD of 1.08 Å and a sequence homology of 34% identical and 53% similar amino acid sequences.

### TrpRS from Encephalitozoon cuniculi bound to L-tryptophan

**Figure 4:**
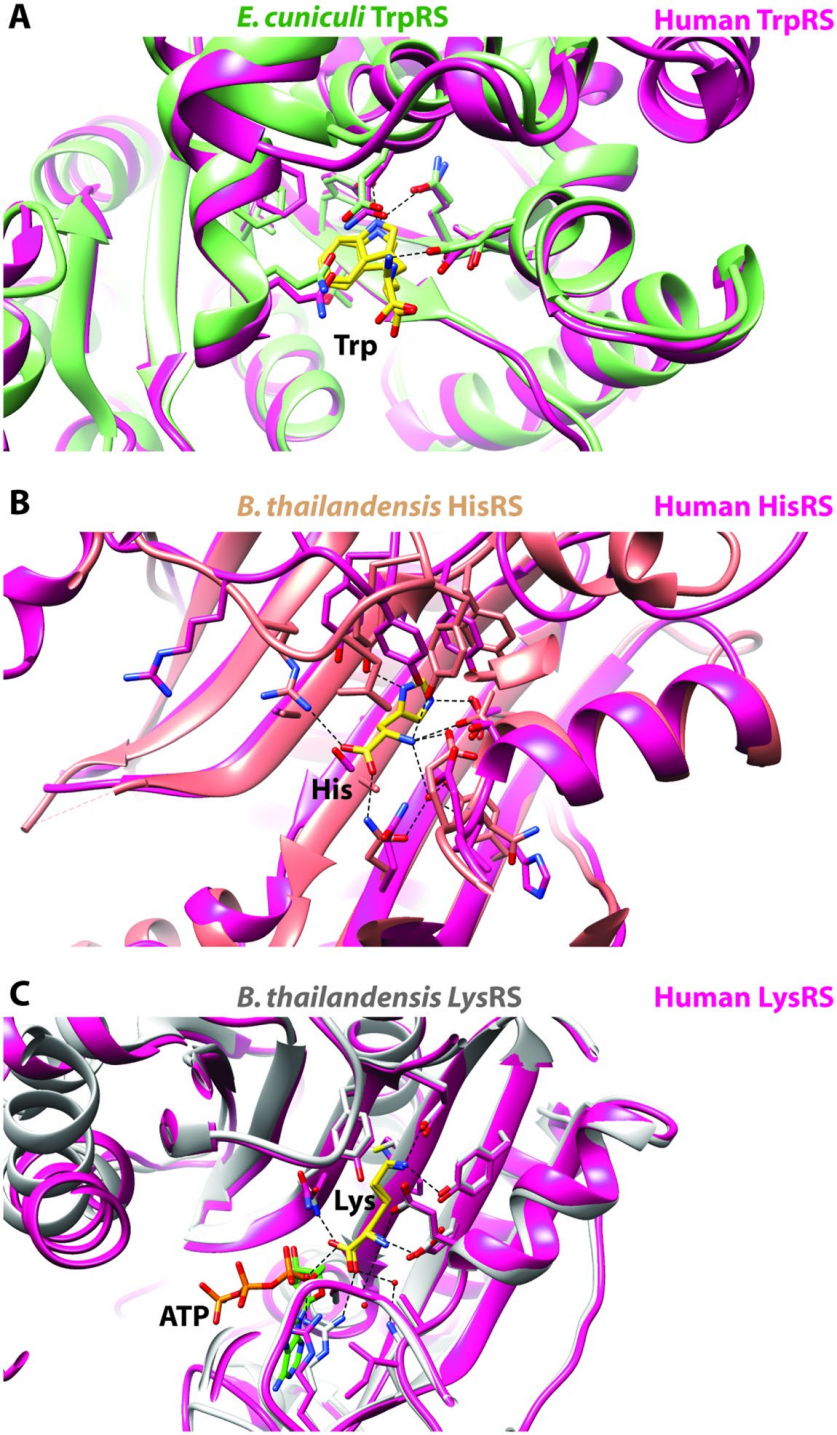
Comparison of the active sites and cognate ligand recognition between aaRSs from human and infectious disease organisms. (**A**) Overlay of *E. cuniculi* TrpRS (PDB ID 3TZE) showing the cognate amino acid binding pocket with human TrpRS (2QUH)^36^ also containing the cognate amino acid, (**B**) *B. thailandensis* HisRS (4E51) showing the cognate amino acid binding pocket with human HisRS (4 × 5O)^39^ which lacks the cognate amino acid, (**C**) *B. thailandensis* LysRS (4EX5) showing the cognate amino acid binding pocket with human LysRS (3BJU)^40^ also containing the cognate amino acid and an ATP molecule.

Crystal structures have been reported for human^36^, yeast^37^, eukaryotic pathogens^22, 25^ as well as bacterial^38^ tryptophanyl-tRNA synthetase (TrpRS E.C. 6.1.1.2) and structures have been reported for human TrpRS/tRNA^Trp^ (2AKE, 2DR2)^36^. We solved a 2.6 Å resolution crystal structure of TrpRS, a class 1c aaRS, from the eukaryotic pathogen *E. cuniculi* with its cognate amino acid L-tryptophan (Figs 1D and 2D). The *E. cuniculi* TrpRS structure was solved during the second round of crystallization trials for this target, as detailed above. The L-tryptophan-bound human (2QUH)^36^ and *E. cuniculi* TrpRS structures are fairly similar and have an RMSD of 1.13 Å between the two structures. The sequence homology between the *E. cuniculi* and human proteins are 46% identical and 65% similar amino acids. Comparison of the TrpRS structures from *E. cuniculi* (3TZE) and human (2QUH)^36^ demonstrates that the same three acids, Glu124, Gln119, and Tyr84 make the same interactions with the cognate amino acid in both structures (Fig. 4A). These three residues make up the only hydrogen bonding interactions of the binding pocket in both structures.

### HisRS from Burkholderia thailandensis bound to L-histidine

A number of histidyl-tRNA synthetase (HisRS E.C. 6.1.1.21) crystal structures have been reported in the literature, including examples from human (4 × 5O)^39^, bacteria (2EL9; no primary citation), and an eukaryotic pathogen (3HRI)^23^. We solved a 2.65 Å resolution structure of HisRS, a class 2a aaRS, from the gram-negative bacteria *B. thailandensis* bound to its cognate amino acid L-histidine (Figs 1E and 3A). *B. thailandensis* is commonly used as a model for *B. pseudomallei* because of their genetic similarity and its far less pathogenic nature. A comparison of the human and the *B. thailandensis* HisRS structures reveals a backbone RMSD of 1.13 Å. The sequence homology between these two proteins is 24% identical and 42% similarity of amino acids. Unfortunately, the human structure is an apo protein so we can only speculate as to the similarities of the binding pocket residue interactions for the human protein (Fig. 4B) but we see homologous human residues for Tyr269, Tyr270, Thr92 and Glu90 that likely play a role in hydrogen bonding of the cognate amino acid in the human protein much like they do in the *B. thailandensis* HisRS structure (Fig. 2).

### LysRS from Burkholderia thailandensis bound to L-Lysine

Crystal structures have been reported for Lysyl-tRNA sythetase (LysRS E.C. 6.1.1.6) from eukaryotic (including human, 3BJU)^40^ and bacterial organisms. We solved a 2.4 Å resolution structure of LysRS, a class 2b aaRS, from *B. thailandensis* bound to L-lysine (Figs 1F and 3B). The cognate amino acid binding pockets of the *B. thailandensis* and human structures are very similar (Fig. 4C) and make many of the same hydrogen bonding interactions. The overall structures of *B. thailandensis* structure reported here (4EX5) and the human LysRS structure (3BJU) is an RMSD of 0.91 Å. These two structures have protein sequence homology of 39% identical and 55% similar amino acids. Recently, several groups have been interested in the inhibitor cladosporin and have solved crystal structures of cladosporin bound to lysyl-tRNA synthetases from *Cryptosporidium parvum* (PDB ID 4ELO; no primary citation), *Loa loa* (PDB ID 5HGQ)^41^, *Plasmodium falciparum* (PDB ID 4YCV)^42^.

## Conclusion

In Fig. 1 the six protein structures of the aaRSs are oriented with the aminoacylation domain up, and the anticodon tRNA binding domain, down. The differences in the overall folds of the aminoacylation domains are apparent for the class I aaRS enzymes that have a Rossman fold (Fig. 1A–D) in comparison with the class II aaRS enzymes which have an anti-parallel β-sheet (Fig. 1E,F). As mentioned earlier, the cognate amino acid binding pocket differences between comparable human structures are subtle. For example, in the *E. cuniculi* TrpRS crystal structure the three residues that make hydrogen bonds with the cognate amino acid, Glu124, Gln119, and Tyr84, overlay almost exactly with the human structures homologous residues. Any compound that would have selectivity between these two proteins would need to utilize more than just these three amino acids in the aminoacyl binding pocket to gain selectivity. Differences, especially just outside the aminoacyl binding pocket, need to be taken advantage of when trying to gain selectivity with a molecular probe compound or potential lead compound. Koh CY, *et al.* use the *T. cruzi* HisRS and build compounds from a site just adjacent to the aminoacyl binding pocket that utilize a cysteine residue found in the *T. cruzi* structure, but not in the human one to develop compounds that are covalent binders^17^. Along similar lines, a number of ProRS inhibitors have been identified with high specificity for pathogenic ProRS enzymes over human enzymes, and these inhibitors such as TCMDC-124506 or glyburide largely bind outside the aminoacyl binding pocket^19^. In addition to the MetRS compounds mentioned above, there are natural products that target other aaRSs (Febrifugine), which might lend more confidence to aaRSs being a viable antibiotic target for some of the organisms discussed in this manuscript. Additionally, there are aaRS inhibitors in clinical trials (Halofuginone) that also make the whole class of aaRSs an interesting group of enzymes from a therapeutic approach. Another clinically relevant aaRS inhibitor, tavaborole, is a topical antifungal medication that inhibits leucyl-tRNA synthetases in onychomycosis fungal infections. The field of aaRS inhibitors has been validated as useful targets for the development of therapeutic compounds; we hope our work will lead to inhibitors against the organisms discussed here. Ideally, these six structures can help guide the creation of more inhibitors and subsequent structures from other organisms.

## Methods

### Protein expression and purification

Detailed SSGCID cloning, protein expression, and purification protocols have been reported previously^43, 44^. Briefly, SSGCID targets were cloned from genomic DNA into an expression vector (pAVA0421) encoding an N-terminal histidine affinity tag followed by the human rhinovirus 3C protease cleavage sequence (the entire tag is MAHHHHHHMGTLEAQTQGPGS). All SSGCID targets were forward and reverse sequence verified. Proteins were expressed in *E. coli* using BL21 (DE3) R3 Rosetta cells and auto-induction media in a LEX bioreactor. The cells were pelleted, frozen at −80 °C. Cells were re-suspended in lysis buffer, sonicated, and clarified by centrifugation. The proteins were purified initially by immobilized metal affinity chromatography. The affinity tag was removed by cleavage with 3C protease followed by a subtractive nickel affinity column for about 60% of all protein samples. For BobuA.00133.a (CysRS), ButhA.00063.a (HisRS), ButhA.00612.a (LysRS), and EncuA.00600.a (TrpRS) that resulted in crystal structures, the expression and affinity tag was not removed prior to crystallization. For ButhA.01187.a (GluRS) and BobuA.01348.a (GluRS) the affinity tag was not removed. All protein samples were further purified, as a polishing step for crystallography, by size exclusion chromatography equilibrated in 20 mM HEPES pH 7.0, 300 mM NaCl, 2 mM DTT, and 5% glycerol. Fractions containing pure protein were collected, pooled, concentrated to ~20–30 mg/ml, and stored at −80 °C prior to crystallization experiments.

### Crystallization

Crystallization trials were set up using the CryoFull, JCSG+, Morpheus, PACT, Synergy, Wizard Full (I/II), and Wizard III/IV sparse matrix crystallization screens from Rigaku Reagents and CSHT, Index, and Salt Rx from Hampton Research. Sitting drop vapor diffusion crystallization trials were set up at 16 °C using 0.4 µL of protein and 0.4 µL of precipitant against 80 µL of reservoir in Compact Jr 96-well crystallization plates from Rigaku Reagents. CysRS from *Borrelia burgdorferi* (BobuA.00133.a) crystallized in the presence of 25% PEG 3350 and 0.2 M Na/K tartrate from the PACT screen condition E9. Both GluRS from *Borrelia burgdorferi* (BobuA.01348.a) supplemented with 20 mM glutamic acid and TrpRS from *Encephalitozoon cuniculi* (EncuA.00600.a) crystallized in the presence of 20% PEG 3350 and 0.2 M Potassium Nitrate from the Wizard III/IV screen condition A8. HisRS from *Burkholderia thailandensis* (ButhA.00063.a) supplemented with 5 mM L-histidine crystallized in the presence of 350 mM Mg Formate, 12% PEG 3350 from a Rigaku Reagents E-Wizard optimization screen from the initial Wizard III/IV screen condition A3 hit. LysRS from *Burkholderia thailandensis* (ButhA.00612.a) crystallized in the presence of 10% PEG 20,000, 20% PEG 550 MME, 0.1 M MOPS/Hepes pH 7.5, 0.02 M of DL-alanine, L-glutamic acid, glycine, DL-lysine and DL-serine from the Morpheus screen condition H5. GluRS from *Burkholderia thailandensis* (ButhA.01187.a) crystallized in the presence of 0.1 M MES/Imidazole, 12.5% PEG 1000, 12.5% PEG 3350, 12.5% MPD, 0.02 M L-glutamate, alanine, lysine, serine, glycine from the Morpheus screen condition H4. Crystals were typically cryo-protected with crystallization reservoir supplemented with 10–25% ethylene glycol or 20% glycerol for ButhA.00063.a and flash frozen by plunging into liquid nitrogen. ButhA.00612.a (Morpheus H5) and ButhA.01187.a (Morpheus H4) were flash frozen without supplemental cryo-protectant.

### Data collection and structure determination

Data sets were collected (Table 2). Diffraction images are available (http://www.csgid.org/csgid/pages/diffraction_images). Molecular replacement was performed using PHASER^45^ from the CCP4 suite^46^. The structure of CysRS from *B. burgdorferi* (BobuA.00133.a) was solved using the structure of CysRS from *E. coli* (PDB ID 1LI5^33^, 33% sequence identity) as a search model. The structure of GluRS from *B. burgdorferi* was solved using 1J09^47^ as a search model. The structure of GluRS from *B. thailandensis* was solved using 4GRI as a search model. The crystal structure of TrpRS from *E. cuniculi* (EncuA.00600.a) was solved using human TrpRS (PDB ID 1ULH^48^, 46% sequence identity) as a search model. The structure of HisRS from *B. thalandensis* (ButhA.00063.a) was solved using HisRS from *E. coli* (PDB ID 1HTT^49^, 55% sequence identity) as a search model. The structure of LysRS from *B. thalandensis* (ButhA.00612.a) was solved using LysRS from *E. coli* (PDB ID 1BBU^50^, 58% sequence identity) as a search model. Structures were built using automated building in BUCCANEER^51^ followed by numerous iterative rounds of manual rebuilding in Coot^52^ and refinement in REFMAC5^53^ or Phenix.Refine^54^. The correctness of each structure was examined, validated, and improved using Molprobity^55^.
